# Embodied Emotion Modulates Neural Signature of Performance Monitoring

**DOI:** 10.1371/journal.pone.0005754

**Published:** 2009-06-01

**Authors:** Daniel Wiswede, Thomas F. Münte, Ulrike M. Krämer, Jascha Rüsseler

**Affiliations:** 1 Department of Neuropsychology, Otto-von Guericke Universität Magdeburg, Magdeburg, Germany; 2 Department of Psychosomatic Medicine, University of Ulm, Ulm, Germany; 3 Center for Behavioral Brain Science, Magdeburg, Germany; 4 Hanse Institute for Advanced Study, Delmenhorst, Germany; James Cook University, Australia

## Abstract

**Background:**

Recent research on the “embodiment of emotion” implies that experiencing an emotion may involve perceptual, somatovisceral, and motor feedback aspects. For example, manipulations of facial expression and posture appear to induce emotional states and influence how affective information is processed. The present study investigates whether performance monitoring, a cognitive process known to be under heavy control of the dopaminergic system, is modulated by induced facial expressions. In particular, we focused on the error-related negativity, an electrophysiological correlate of performance monitoring.

**Methods/Principal Findings:**

During a choice reaction task, participants held a Chinese chop stick either horizontally between the teeth (“smile” condition) or, in different runs, vertically (“no smile”) with the upper lip. In a third control condition, no chop stick was used (“no stick”). It could be shown on a separate sample that the facial feedback procedure is feasible to induce mild changes in positive affect. In the ERP sample, the smile condition, hypothesized to lead to an increase in dopaminergic activity, was associated with a decrease of ERN amplitude relative to “no smile” and “no stick” conditions.

**Conclusion:**

Embodying emotions by induced facial expressions leads to a changes in the neural correlates of error detection. We suggest that this is due to the joint influence of the dopaminergic system on positive affect and performance monitoring.

## Introduction

**Figure 1:**
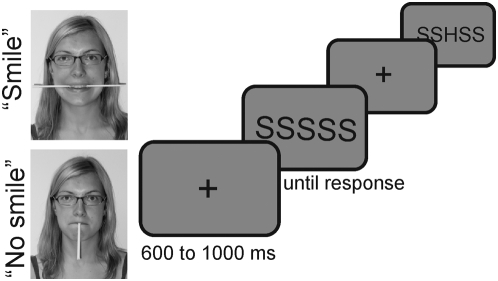
Experimental setup. The photos show the typical facial expressions induced by the chop stick in the smile and no-smile conditions.

It has been shown that people who are adopting an emotion-specific posture report to experience this emotion [1], show behavior congruent with the emotion [2], or show emotion-specific changes in autonomic nervous system activity [3]. For example, people rate cartoons to be funnier when they have a pen between their teeth in a way that leads to contraction of the musculus zygomaticus major, a muscle essential for smiling [2], compared to a control condition requiring to hold the pen vertically between the lips. The latter posture prevents participants from smiling. Similarly, Havas, Glenberg, & Rinck [4] observed that the amount of time to judge the valence of a sentence is influenced by the kind of emotion that is induced from holding a stick in the mouth. For both positions of the stick in the mouth (between the teeth, i.e. smiling, between the lips, i.e. frowning) judgment times were faster when facial posture and sentence valence matched than when they were incongruent. Intriguingly, people are not usually aware that they are smiling [2]. This excludes alternative explanations based on people's self-perception, for example that people perceive themselves to be smiling and infer to be happy. However, one might argue that people are set in an emotional state, because they feel silly or funny when holding a pen in the mouth during an experiment. Strack and colleagues [2] elegantly excluded this alternative by introducing the “hold the pen with the lips” condition (see figure 1 for assumed facial expression): here, participants are prevented from smiling, but there is no reason to assume that they feel less silly or funny compared to the “pen between the teeth” condition. All in all, these findings suggest that by assuming a facial expression of a body posture, the corresponding affect is induced. This “embodying of emotion” [1] is thought to be brought about by the fact that reinstantiation of an activation pattern in one system (e.g., facial muscles typically active when “happy”) can cascade down to other systems to install the full activation pattern associated with the particular emotion. In some sense the recent interest in embodying of emotions echoes the classical work by James [5] and Lange [6]. In his 1884 paper, James [5] ascertained that a “*mental state is not immediately induced… [but] that the bodily manifestations must first be interposed between, and that the more rational statement is that we feel sorry because we cry, angry because we strike, afraid because we tremble, and not that we cry, strike, or tremble, because we are sorry, angry, or fearful, as the case may be. Without the bodily states following on the perception, the latter would be purely cognitive in form, pale, colourless, destitute of emotional warmth.”*

The question arises how this embodiment of emotion in the sense of James [5] and, more recently, Niedenthal [1] is brought about. Importantly, recent data on the so-called mirror neuron system (MNS) have pointed to a role of this system in social cognition [7]–[10] and more specifically in emotion recognition [11]. The MNS has been first identified in monkeys: mirror neurons located in the inferior frontal cortex but also in a number of other brain areas fire not only when an action is performed by the monkey but also when the same action is observed. Recently, fMRI studies in humans have shown a relation of brain activity in regions harboring mirror neurons and an individual's empathic feelings [12]–[14] likely because activity of the MNS allows the recognition of an emotion. The link to the aforementioned literature on embodied emotions is provided by studies that have implied the MNS in the spontaneous mimicry of emotional facial expressions [15] in order to internally simulate the perceived emotion and to aid its understanding. For example, the prevention of facial mimicry impairs the detection of a change in emotional facial expressions [16]. Another crucial experiment was reported by Oberman et al. [17]. These authors tested recognition of facial expressions (happy, disgust, fear, sad) and blocked mimicry by having their participants either bite on a pen or chew a gum. The bite manipulation in particular interfered with the recognition of happiness suggesting that assuming a facial expression is necessary for its recognition and, by extension, its experience.

In the present investigation we go a step further by examining the influence of assumed facial expressions thought to induce positive affect on performance monitoring. Indeed, positive affect makes people react differently. There is accumulating evidence that positive affect facilitates problem solving [7], [8], memory performance [9], executive attention [10], and a variety of other cognitive task. Ashby and colleagues [11] argue that positive affect is associated with an increased brain dopamine level in a variety of dopaminergic structures, among them the mesocorticolimbic system, prefrontal cortex and anterior cingulate cortex. These structures are involved in reward and reward prediction (e.g., the ventral tegmental area, which is highly interconnected to the Nucleus Accumbens) [12]–[15] as well as cognitive control [16]–[18]. The mesencephalic dopamine structures and their interactions with the prefrontal cortex are also central in research on performance monitoring, which includes the detection and correction of errors and the adaptation of behavioral strategies to minimize errors in subsequent trials. According to the reinforcement learning hypothesis of error processing [19], inspired by earlier work on animals [20], [21], error commission results in decreased activation of the mesencephalic dopamine system. This, in turn, leads to a phasic disinhibition of the anterior cingulate cortex, which is reflected by brain activation to error trials in choice reaction time tasks [22]–[24] as well as by an increased negative amplitude of event-related brain potentials (ERP). Specifically, when ERPs are obtained time-locked to choice errors, an “error related negativity” (ERN, sometimes also Ne, for error negativity) emerges [25], [26] which onsets around the commission of the error and peaks around 100 ms with a medio-frontocentral maximum.

The ERN can be modulated by motivational and emotional factors. It is increased in participants scoring high on scales for anxiety and worry [27], [28], in participants suffering from obsessive-compulsive disorder who often have comorbid depressive symptoms [29]–[31] and after presentation of negative IAPS pictures [32], [33]. In contrast, if and how positive emotions influence performance monitoring is not known. Possible indirect evidence comes from drug studies but has to be regarded with caution: Alcohol, which induces pleasant feelings, and oxazepam, a benzodiazepine derivative with anxiolytic properties, reduce ERN amplitude [34], [35].

The present study therefore examines how induced facial expressions modulates ERN amplitude. Following Ashby and colleagues [11], we hypothesized that induced smiles (positive affect) increases dopaminergic activity in various brain regions, among them the mesencephalic dopamine system and the prefrontal cortex. This increase in dopaminergic activity should offset the phasic decrease in this neurotransmitter induced by performance errors and, hence, we expected a decreased ERN amplitude in a smile vs. a no-smile condition. Normal participants were studied in a typical flanker experiment (see figure 1) in three conditions (stick between the teeth: “smile”, stick held with the upper lip: “no smile”, control “no stick”). Since participants were to remain naïve regarding the intended emotional modulation, the effectiveness of the facial feedback procedure was tested in a separate sample.

## Results

### Induction of emotion

**Figure 2:**
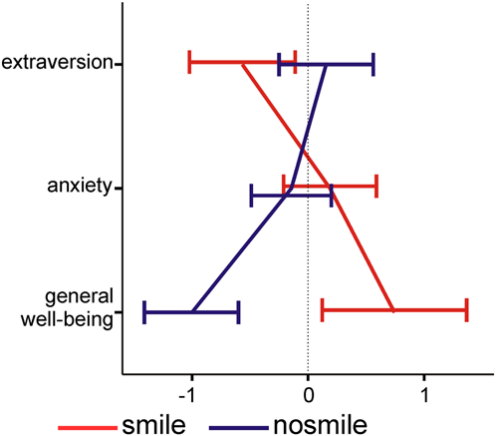
Results of the EWL-60-S questionnaire; Data were collected on a separate sample, Facial Feedback group is coded by line color. Items summarized according to the EWL-60-S-manual. Scores are based on difference post-experimental EWL-60-S minus pre-experimental EWL-60-S results; positive values indicate that people loaded higher on this scale after completion of the EEG experiment. Error bars indicate +/−1 SE.

- In the behavioral sample: After the experiment, subjects of the “non-smile” condition scored lower on the EWL-60-S-scale “general well-being”, whereas subjects of the smile condition scored higher (t(28) = 2.3, p<0.03). There were no differences on the scales “extraversion/introversion” and “anxiety” (figure 2). Gender differences could not be examined in detail due to the limited number of male subjects in the sample.
- In the ERP sample: No formal assessment of induced mood was conducted to leave the participants naïve with regard to the emotion induction manipulation. After the experiment, participants were asked informally whether they felt differently with varying stick positions. While three participants reported that they felt “positive”, “good” or “happy” in the “smile”-condition, no such response was obtained in the “no smile”-condition.

#### Behavioral data

Reaction times and error rates are given in table 1. Erroneous responses were faster than correct responses, but the three facial feedback conditions did not influence reaction times (*correctness*: F (1,21) = 339.4, p<.001; *expression: F (2,42) = 2.3, p<.11,* interaction: *F (2,42) = 1.45, p<.24*). Responses were faster following congruent flankers (HHHHH and SSSSS) compared to incongruent flankers (HHSHH and SSHSS) but the congruency effect was not modulated by *expression* (*congruency*: F (1, 21) = 265.1; p<.001; *expression: F (2,42) = .87, p<.42; congruency by expression: F (2,42) = .78, p<.45*). However, facial feedback modulation had a small, but significant impact on error rates (*congruency*: F (1, 21) = 251.95; p<.001; *expression: F (2,42) = 4.7, p<.014; congruency×expression: F (2,42) = 2.47, p<.10*) mean error rates: congruent flanker: 5.6%; incongruent flanker: 18.9% see table 1 for detailed error rates. However, post hoc comparison revealed that the difference was seen between the smile and the no-stick condition (t = 3.2; p <.005). There were no differences between the smile and the non-smile condition.

**Table 1:** Reaction times and error rates.

		overall	smile	no-smile	no stick
**Reaction times in ms**	**Correct**	395	394	395	397
	**Correct/congruent**	379	377	379	381
	**Correct/incong.**	424	424	423	425
	**Error**	336	333	337	338
**Error rates in percent**	**Errors**	10.9	11.5	10.8	10.4
	**Errors/congruent**	5.6	5.9	5.3	5.6
	**Errors/incongruent**	18.9	19.9	19.1	17.6

Participants showed significant post error slowing (F (1,21) = 28.99, p<.001) but this was not modulated by *expression* (*F (2,42) = 0.61, p<.94*, see table 2).

**Table 2:** Post-error slowing.

Errors	Overall	smile	no-smile	no stick
Postcorrect trials	391	393	392	389
Posterror trials	418	417	416	421

Correct responses following erroneous responses (posterror trials) are compared with correct responses following response-matched correct responses (postcorrect trials). Reaction times are given in milliseconds.

### Response-locked ERPs

**Figure 3:**
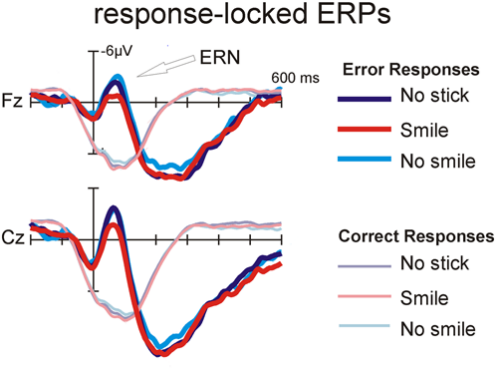
Response-locked ERPs on midline electrodes FZ and CZ for correct (thin lines) and erroneous (thick lines) responses. Stick-positions are coded by line style.

**Figure 4:**
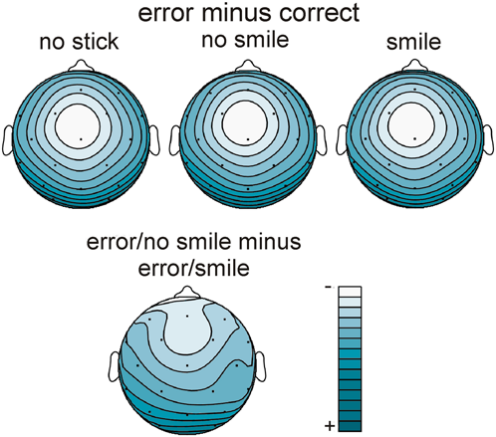
Spline-interpolated isovoltage maps depicting the mean amplitude in a time window 20 to 100 ms of the difference wave “error” minus “correct”. Relative scaling is used with lighter shades representing more negative amplitudes.

Response-locked averages showed a typical ERN response that peaked at about 70 ms post stimulus (figure 3) and was most pronounced over the medial frontal scalp (electrodes Fz, Cz, FC1, FC2). Visual inspection indicated a considerably smaller ERN amplitude in the smile condition, whereas the ERPs to the correct stimuli were not modulated by facial expression. Moreover, spline interpolated isovoltage maps of the ERN did not reveal differences in scalp distribution between conditions (see figure 4).

Statistical analysis was conducted separately for correct and erroneous responses (mean amplitude 20 to100 ms; Fz, Cz, FC1, FC2). The reduced ERN for the smile condition led to a main effect of expression for the error trials (F (2,42) = 5.20; p<.01), with post-hoc comparisons showing that the smile condition was different from both, the no-smile and no-stick condition (both p<0.05). No significant effect of *expression* was seen for correct trials (F (2,42) = 1.76; p<.19).

## Discussion

Inducing a smiling facial expression by holding a pen between the teeth led to an increase in general well-being (behavioural sample) and to an increase in error rate accompanied by a reduction of the error-related negativity (ERN), a prominent neurophysiological marker of performance monitoring. Thus, the experimental manipulation was successful with regard to our main target of observation and the direction of amplitude change conformed to our expectations derived from the reinforcement learning theory of the ERN [19]. This theory specifies that error detection involves the dopaminergic midbrain and that a performance error is associated with a phasic decrease of dopamine that is transmitted to the medial prefrontal cortex, where the ERN is released. Recent invasive measurements corroborated this account by showing that error-related activity is also present in the Nucleus accumbens, a structure heavily innervated by dopaminergic activity [31].

In their model of dopamine and positive affect, Ashby and coworkers [11] propose that positive affect leads to an increased dopamine release in the midbrain (nigrostriatal and mesocorticolimbic system) and in frontal brain regions. Thus, inducing positive mood elevates the tonic dopamine level. Following work on the embodying of emotion [1], [2], [4] and in particular investigations examining the facial feedback hypothesis [36]–[40] we assume that our manipulation led to mildly positive affect. As a consequence, the dopamine level in the anterior cingulate cortex (ACC) should be tonically increased [11]. Increased dopamine levels in the midbrain and in the ACC due to the induced positive affect might counteract the phasic reduction in dopamine activity [19] and cause a weaker disinhibition of the ACC. This might be the neural mechanism that leads to the decreased ERN-amplitude we observed in the “smile”-condition of the current experiment. Ashby and Casale [41] proposed a computational model that simulates the tonic dopamine increase by positive affect. They showed that their model is able to generate results like the one we obtained here by simply changing the numerical value of the two parameters assumed to be affected by the dopamine level (parameters K_ampa_(Da) and K_e_(Da) in the model).

The present study supplements earlier findings from our group [32], [33] where short-term presentation of negative IAPS-pictures prior to the execution of a flanker task lead to an increase in ERN-amplitude. In our earlier work, positive IAPS-pictures presented immediately prior to the flanker stimuli did not lead to a change in ERN-amplitude. This might be explained by the computational model mentioned above [41], according to which positive affect must last at least 30 s to affect dopamine level.

The clear reduction of the ERN amplitude in the smile condition was accompanied by an increase in error rates, which fits with previous notions that positive affect is associated with decrements in performance quality [42], [43]. By contrast, facial expression had no influence on other behavioral measures in the flanker task such as reaction times and post error slowing. A dissociation of behavioral measures and ERN changes has been reported repeatedly (e.g. [27]–[29]) and it remains to be shown whether a more profound positive mood change would affect reaction times and post-error slowing in addition to error rates.

The present ERP experiment involved only female participants. Several lines of evidence indicate that emotional expressions might differ between male and female. For example, it has been shown that woman generate facial electromyographic pattern of greater magnitude and report stronger experience of emotion while imagining emotional situations [44]. Thus, if emotional experience corresponds more strongly to facial expressions in women, manipulation of facial expression might also lead to a stronger emotional response in women. Others have argued, however, that facial feedback might be more powerful in men than women, because the former might be more sensitive to physiological changes [45]. Facial feedback effects have been previously described in a female only sample [46]. Thus, it remains to be explored whether the ERN effects might be generalized to a male population as well.

To sum up, the present study for the first time shows that induced facial expressions known to lead to positive affect leads to reduced activity of the performance monitoring and error detection system. Previous research has shown that positive affect may lead to increased cognitive flexibility [42], [43]. The present results are compatible with this earlier finding: positive affect might lead to less emphasis on error avoidance and thus allow the subject more flexible behavior.

## Materials and Methods

### Participants

ERP study: Twenty-five right-handed women took part in the experiment. Data from 3 participants had to be excluded (two participants due to high and uncorrectable artifact levels, one participant mixed up stick-positions (see below)). Thus, data were analyzed from 22 women (mean age 22 years, range 17 to 28) all having normal or corrected to normal vision. As previous research on mood induction (for example using pictures with emotional content) has revealed gender differences and more pronounced effects in women, we included only female participants in the ERP study. Please note that this also ensured comparability with previous research on emotional modulation of the ERN [32], [33].

Behavioral sample: There was a separate sample to assess changes in short-term psychological state, consisting of 30 participants (21 female, mean age 25, range 19 to 53).

Participants received course credit or €6.50 per hour after completion of the experiment and gave written informed consent. The study protocol was approved by the ethics committee of the University of Magdeburg.

### Stimuli and Procedure

#### Flanker-task

A trial consisted of the following sequence, timing is provided in brackets: fixation cross (600 to 800 ms, mean 700 ms), flanker stimulus until response (see Fig. 1). Flanker stimuli consisted of black capital letters (“Courier new” font) H or S presented in front of a gray background (128, 128, 128 in RGB color space). A congruent flanker string was either HHHHH or SSSSS; incongruent flanker strings were SSHSS or HHSHH. Flankers were presented in random order. There were 60% congruent and 40% incongruent trials. They covered 2.1° of visual angle in width. Participants were asked to respond as fast and as correct as possible to the central letter of the flanker string. They responded with a left-hand button to the H and with a right-hand button to the S.

The experiment consisted of 2100 trials. A feedback screen was presented after every 30 trials, informing the participants whether they had been faster or slower than in the previous 30 trials. This procedure was introduced to keep participants attending and to maintain fast responses. Participants terminated the feedback screen by button press. After 210 trials (1 block), there was a break for 15 seconds. Participants could request longer breaks if necessary.

#### Induction of facial expression

Affective state was modulated on a block-wise basis. At the beginning of each block, participants were asked to either

- hold a Chinese disposable chopstick horizontally between the teeth (“smile”-condition),
- hold a stick vertically with the upper lip only (“no-smile”-condition) or
- have no stick in the mouth (“no stick”-condition).

Three blocks of each condition were performed in a quasi-randomized manner (restriction: the same condition could not be performed in two successive blocks). Thus, 3×210 = 630 trials were obtained for each condition.

#### Sham story

Participants were not informed about the intended affective modulation. Thus, to explain why they were required to hold a chopstick with either the lips or between the teeth, the following sham story was introduced: Participants were told that the present ERP study examines how facial muscle artifacts influence ERP recordings. To demonstrate muscle artifacts, the experimenter presented the participants with their own EEG on the presentation computer prior to the beginning of the experiment. Participants were asked to blink and to move the eyes so that clearly detectable blink artifacts were visible in the EEG-tracings. Now that participants knew that eye artifacts severely impact ERP recordings, they were told that researchers know how to handle eye artifacts, but that little is known about how to handle muscle artifacts generated by the mouth and ervated by different chop stick positions. At the end of the experiment, a questionnaire asked the participants to explain the purpose of the experiment. None of them was suspicious about the cover story. After the completion of the experimental session, participants were debriefed.

#### Assessment of emotional state

Since it was intendedby the cheek muscles. Thus, to examine this in detail, mouth and cheek muscles will be inn to leave the participants naïve regarding the emotion induction procedure, it was necessary to test the effectiveness of the facial feedback procedure on a separate sample. Thirty subjects, all participants of another ERP study in our lab, were asked to fill out the EWL-60-S [47], [48] after completion of the ERP setup procedure. The EWL-60-S is an established German questionnaire to assess short-term changes in psychological state; it is a shortened version of the German adjective list “Eigenschaftswörterliste” [48] and well-suited to before and after treatment [47]. The EWL-60-S consists of 60 items, summarized to 6 scales. In the present study, we restricted to the scales “extraversion/introversion” (in this context, extraversion is not treated as a personality trait, but refers to a person's present mood state [49]), “general well-being” and “anxiety”, each consisting of two subscales and 8 items. After completion of the questionnaire, participants received the same sham story as the ERP sample of the current experiment. They were randomly allocated to hold the chopstick for five minutes either in the “smile” position or in the nonsmile position. After that, they filled out a rearranged version of the EWL-60-S.

### Data recording and analysis

Recordings were conducted in an electrically shielded recording chamber equipped with a Neuroscan EEG amplifier. Participants were seated in a comfortable chair at a distance of 80 cm to the screen. Stimuli were presented on a 19 inch analog monitor. Chamber illumination was slightly dimmed.

The electroencephalogram (EEG) was recorded from 29 positions including all 19 standard locations of the 10/20 system with tin electrodes mounted in an elastic cap relative to a reference electrode placed on the tip of the nose. Eye-movements were recorded with electrodes affixed to the right and left external canthi (horizontal electrooculogram (hEOG), bipolar recording) and at the left and right orbital ridges (vertical electrooculogram (vEOG), bipolar recording). Impedances of all electrodes were kept below 10 kΩ. Biosignals were amplified with a band-pass from 0.05 to 30 Hz and stored with a digitization rate of 250 Hz. Prior to ERP data analysis, all trials containing eye artifacts were corrected using a blind component separation [50]. Artifacts on recording channels were rejected based on individual peak-to-peak amplitude criteria using a special purpose program with individual thresholds between 50 and 100 µV. Stimulus-locked ERPs (onset of emotional picture and onset of flanker stimulus) were averaged for epochs of 1024 ms starting 100 ms prior to stimulus onset for stimulus-locked data analysis and 200 ms prior to response for response-locked analysis. The pre-stimulus period served as a baseline for ERP-computation. All ERP figures and all ERP statistics are based on unfiltered data (except band-pass from 0.05 to 30 Hz during recording).

ERPs were generated relative to a 200 ms pre-response baseline. Consistent with previous research [51], only responses given within 200 to 800 ms after flanker stimulus onset were included in ERP analysis and behavior data. Statistical analysis was based on the factors *correctness* (correct vs. erroneous responses) and *expression* (stick position; “smile”, “no smile”, “no stick”). The ERN was quantified by a mean amplitude measure (20–100 ms) for frontocentral electrodes (averaged across electrodes FC1, FC2, Fz, Cz).

Reaction times (only reactions given in a 200–800 ms post stimulus window) and error rates (percentage) were obtained and entered into ANOVA statistics. We also examined post error slowing. This term refers to the fact that often correct responses directly following an erroneous response (post-error trials) are slower relative to trials that follow correct responses (post-correct trials; e.g. [52], [53]). However, since responses for erroneous trials are usually faster than for correct trials, this effect could be caused by regression toward the mean. As fast responses are relatively rare, it is more likely that a fast response is succeeded by a slower response. To distinguish between post-error effects caused by regression towards the mean from “pure” error-induced RT slowing, a subset of correct trials was selected that matched the erroneous trials in terms of reaction time and total number (see [27] for a similar procedure). Thus, the selected correct trials belong to the faster responses among all correct trials. Reaction times of correct trials given directly after those response-matched correct trials (post-correct trials) and response times of correct responses given directly after an erroneous response (post-error trials) provide the basis for post-error slowing analysis.

Emotional state in separate sample: EWL-60-S-scales were compared between the “smile” and the “no-smile” group via independent t-tests based on the difference post-treatment-score minus pre-treatment-score.
